# The role of steroid injection in joints and tendon sheaths in JIA in the biologic era

**DOI:** 10.1186/1546-0096-12-S1-P185

**Published:** 2014-09-17

**Authors:** Virginia Messia, Denise Pires Marafon, Fabrizio De Benedetti, Silvia Magni-Manzoni

**Affiliations:** 1 Pediatric Rheumatology, Ospedale pediatrico Bambino Gesù, Rome, Italy

## Introduction

Intraarticular injections are commonly used in children with JIA to induce prompt relief of symptoms of active synovitis, and obtain disease remission as sole therapy or in combination with systemic treatment. In the recent years, the wider use of musculoskeletal ultrasound allowed to detect more precisely the exact site of inflammation and showed to be a safe guide for injection procedures also in tendons sheaths and periarticular tissue.

## Objectives

The aim of the study was to investigate the role of intraarticular steroid injections and injection of tendon sheaths in the management of children with JIA in a pediatric rheumatology tertiary care center in the recent years.

## Methods

The charts of all the patients with JIA that underwent to steroid injection of joints and/or tendon sheaths from January 2012 to April 2014 in our center were reviewed. Demographic and clinical data at the time of the steroid injections were registered, including ongoing therapy and decision to start a new systemic treatment. In the follow-up visits, remission of synovitis in the injected sites was clinically defined as the absence of swelling and/or pain on motion/tenderness associated to limited joint restriction.

## Results

From January 2012 to April 2014 we performed 181 steroid injection sessions in 145 JIA patients. 25 patients (17%) underwent to more than one session: 51 sessions were conducted under local anesthesia, while 130 under general sedation. In 65 sessions only one joint or tendon sheath was injected; 2 injections were performed in 51 sessions; 3 or more injections in the same session were conducted in 75 sessions. The total number of sites injected was 456, 71 of which (16%) were represented by tendon sheaths or cysts. After a median period of 4,9 months 79% of the sites injected were in clinical remission. Of the 21% of sites with persistent synovitis at the first follow-up visit, 50% achieved remission without adding new treatments after a median of 7 months from the procedure, while 25% achieved remission only after the introduction of new systemic treatments; the remaining 25% were not in complete remission at the last follow-up visit yet.

## Conclusion

Intraarticular and tendon sheaths injections in our center have been extensively performed in the recent years for the treatment of synovitis in one or multiple joint areas in children with JIA. Further longitudinal studies including imaging definition of remission should be performed to investigate the long term efficacy of these therapeutic procedures.

## Disclosure of interest

None declared.

